# LongReadSum: A fast and flexible quality control and signal summarization tool for long-read sequencing data

**DOI:** 10.1016/j.csbj.2025.01.019

**Published:** 2025-01-24

**Authors:** Jonathan Elliot Perdomo, Mian Umair Ahsan, Qian Liu, Li Fang, Kai Wang

**Affiliations:** a Raymond G. Perelman Center for Cellular and Molecular Therapeutics, Children’s Hospital of Philadelphia, Philadelphia, PA 19104, USA; b School of Biomedical Engineering, Drexel University, Philadelphia, PA 19104, USA

**Keywords:** Quality control, Long-read sequencing

## Abstract

While several well-established quality control (QC) tools exist for short-read sequencing data, there is a general paucity of computational tools that efficiently deliver comprehensive metrics across a wide range of long-read sequencing data formats, such as Oxford Nanopore (ONT) POD5, ONT FAST5, ONT basecall summary, Pacific Biosciences (PacBio) unaligned BAM, and Illumina Complete Long Read (ICLR) FASTQ file formats. In addition to nucleotide sequence information, some file formats such as POD5 contain raw signal information used for base calling, while other file formats such as aligned BAM contain alignments to a linear reference genome or transcriptome and may also contain base modification information. There is currently no single available QC tool capable of summarizing each of these features. Furthermore, high-performance tools are required to efficiently process the growing data volumes from long-read sequencing platforms. To address these challenges, here we present LongReadSum, a high-performance tool for generating a summary QC report for major types of long-read sequencing data. We also demonstrate a few examples using LongReadSum to analyze cDNA sequencing, direct RNA sequencing, ONT reduced representation methylation sequencing (RRMS), and whole genome sequencing (WGS) data.

## Graphical Abstract

**Figure:**
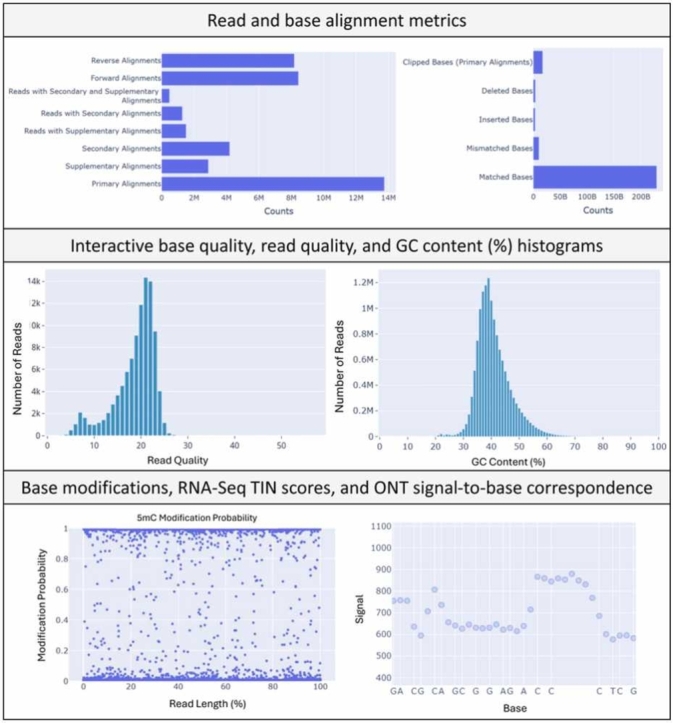

## 1 Introduction

Recent advancements in long-read sequencing technologies, particularly from Oxford Nanopore Technologies (ONT) and Pacific Biosciences (PacBio), allow users to sequence reads that are tens to thousands of kilobases (kb) long with high accuracy. Due to these momentous methodological advancements and their broad applications, long-read sequencing was chosen by the journal *Nature Methods* as “Method of the Year 2022” [1]. In 2023, Illumina officially released their own high-accuracy Complete Long Read technology with an N50 read length of 5–7 kb [2]. These advancements make long read sequencing a viable option for a broad range of applications in genomics, but require the development of novel bioinformatics tools that can quickly summarize important technology-specific metrics for quality control (QC) purposes. QC is a vital component of any sequencing pipeline to identify potential biases and technical artifacts that may affect the downstream analysis and interpretation of results, and to understand basic characteristics of the sequencing run. Long-read sequencing data also has unique characteristics relative to short reads, such as broader read length distributions, which require distinct QC metrics. In addition, these high throughput sequencing platforms require high-performance tools capable of quickly processing large amounts of data: While short reads can produce reads ∼35–700 bases long [3], current long reads are typically in excess of > 10 kilobases [4]. Currently, a single ONT PromethION flowcell may generate up to 290 gigabases of sequence data, with up to 2.6 terabytes of signal data in FAST5 format [5], and similarly for the POD5 format. Thus, long read QC tools must have the scalability to process these growing datasets in a fast manner to ensure that QC can be included as part of a routine sequencing pipeline.

There are several widely used and established short read QC tools such as FastQC [6] that could be used with long read sequencing data. FastQC generates a summary HTML report providing an overview of raw sequence data and supports both BAM/SAM and FASTQ data formats [6]. However, FastQC does not provide read and base mapping QC or important long read metrics such as mean, median, maximum, and N50 read length values, and it has not been optimized to handle large datasets [6]. Thus, there is a need for a tool that can produce fast, high throughput, and comprehensive QC summary statistics for important long read characteristics. In addition, it is often useful to leverage a combination of sequencing technologies to achieve optimal results. These integrative pipelines necessitate a versatile tool that can generate QC metrics across platforms. Nevertheless, currently available QC tools for long-read data such as NanoPlot [7], PycoQC [8], NanoQ [9], NanoQC [7] and MinIONQC [10] typically support sequencing data formats for only one specific platform, such as PacBio or ONT, only support a subset of the major data formats, or only focus on a specific aspect of the data, such as base modifications (Table 1) [7], [8], [9], [10], [11]. In addition, to our knowledge there are currently no available ONT QC tools for visualizing signal intensity and corresponding basecalls from ONT FAST5 and POD5 file formats, or for extracting signals in specific genomic regions, which are important for epigenomics and epitranscriptomics studies.

**Table 1:** Comparison of filetype support vs. available long read QC tools (seqtxt = basecall summary files, uBAM=PacBio unaligned BAM). *For PycoQC, BAM analysis requires the basecall summary file, and FAST5 analysis requires first converting the FAST5 files to basecall summary format using their tool.

**Tool**	**FASTA**	**FASTQ**	**BAM**	**uBAM**	**seqtxt**	**FAST5**	**POD5**
LongReadSum	Y	Y	Y	Y	Y	Y	Y
NanoPlot	Y	Y	Y	Y	Y	N	N
PycoQC	N	N	Y*	N	Y	Y*	N
NanoQ	Y	Y	N	N	N	N	N
NanoQC	N	Y	N	N	N	N	N
MinIONQC	N	N	N	N	Y	N	N

Here we present LongReadSum, a computational tool for generating fast, comprehensive, and high throughput long read QC summaries. It supports all major long-read sequencing technology data formats. To the best of our knowledge, it is also among the first QC tools enabling visualization of ONT signal intensity and basecall information from ONT FAST5 and the latest POD5 file formats.

## 2 Implementation

LongReadSum is run from the command line as a non-interactive Python module, and it generates a comprehensive summary of different aspects of sequencing data in a timely manner by utilizing a flexible multi-threaded C++ framework. All QC metrics are computed in a C++ module and results are compiled into a dynamic HTML report and summary text file using Python. The C++ module is wrapped for Python interfacing using SWIG. We leverage C++ multi-threading for BAM and basecall summary file analysis to achieve higher performance: On a 32-core computer with 4 Intel Xeon E5–4627 V2 3.3 GHz 8 Core Processors with 1TB memory, QC metrics for an aligned BAM file (57 gigabases, with an N50 of 22 kilobases) from a single PromethION flow cell are completed in ∼15 minutes when running on 8 threads. We provide an Anaconda package for quick, single-command installation in Linux systems, as well as a Docker container option for platform-independent installation.

## 3 Supported data formats

LongReadSum provides QC information for all major sequencing data formats, generating filetype-specific results as a summary text file and as an HTML-based interactive report.

### 3.1 FASTA and basecall summary files

**Fig. 1:**
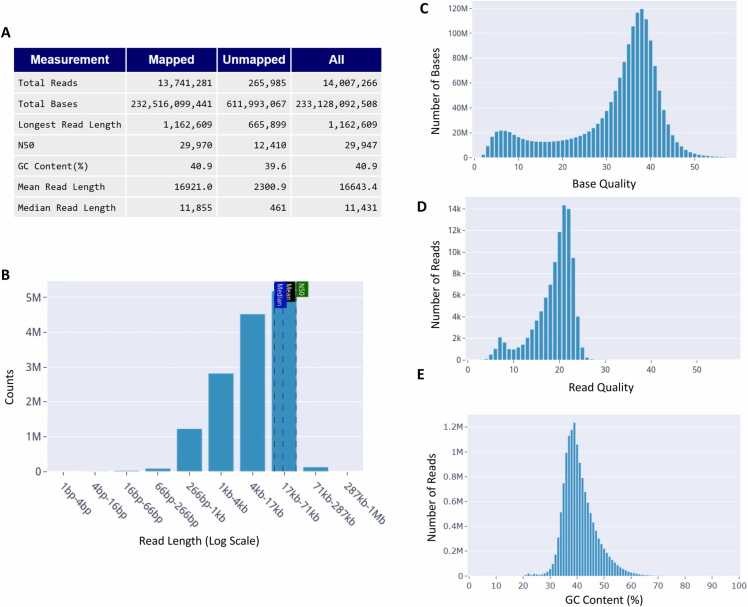
Basic QC metrics from a BAM file of HG002 sequenced with ONT Kit V14 PromethION R10.4.1. (A) Table of read and base metrics. (B) Read length histogram (log scale). (C) Base quality histogram. (D) Read quality histogram. Read quality is calculated by converting per-base Q score to accuracy, taking the average of all bases in a read, then converting it back to a Phred-scaled per-read quality score. (E) GC content histogram across reads.

FASTA is the simplest format containing only a text-based representation of sequence records [12]. This format is commonly used for assemblies which can be generated from long-read sequencing. ONT basecall summary files also contain basic sequencing read information. For data in these formats, the QC report contains basic statistics including the total number of reads, base pairs, maximum, mean, and median read length, N50 (Fig. 1A), and a read length histogram (Fig. 1B). FASTA reports also include base counts for each nucleotide, percent guanine-cytosine (GC) content, and a histogram of per-read GC content distributions (Fig. 1E).

### 3.2 FASTQ, FAST5, and unaligned BAM

The FASTQ, FAST5 and unaligned BAM formats build upon FASTA to include sequencing read base quality information [13] where base quality is represented as PHRED scores reported by the sequencing platform [14], [15]. For these file formats with base quality information, the QC report generates a histogram of base quality (Fig. 1C) and read average base quality distributions (Fig. 1D).

### 3.3 BAM with alignments, base modifications, and/or RNA-Seq data

The BAM file format stores a binary representation of read alignments against a reference sequence [16]. For BAM files with alignment information, the report provides a summary of alignment types and counts at the read and base level (Fig. 4). ONT BAM files may contain base modification information as well. Modkit [11] is an existing tool for generating ONT base modification summary statistics, but it does not provide other measures such as the read length vs. modification probability information (calculated by modification callers such as Dorado [17] and DeepMod2 [18]) for specific types of base modifications), which is useful for identifying biases in modification detection within sequencing reads. In addition to basic statistics, LongReadSum generates additional summary plots for each type of modification, including a scatter plot and histogram of probabilities for modification predictions (Suppl. Fig. 1) and a summary table of modification prediction counts (Suppl. Table 1). For RNA-Seq BAM files, the transcript integrity number (TIN) represents the uniformity of coverage for a given transcript, while the median TIN score across transcripts is an important metric for assessing RNA integrity at the sample level [19]. The method implemented in LongReadSum is adapted from the RSeQC software package [20], which calculates a median TIN score and includes it in a summary text file. LongReadSum produces these outputs in TSV format and includes the TIN median score in addition to the full summary of the BAM file in the HTML report.

### 3.4 FAST5 with basecalls

The ONT FAST5 file stores sequencing reads containing the raw time series signal data for ionic currents and may also contain the basecalled sequence. There are a few currently available tools specifically for FAST5 signal visualization and signal-to-base comparisons, which are useful for identifying signal anomalies in regions of interest [21], [22], [23]. Signal data analysis has already proven useful for short tandem (STR) repeat detection [24], [25], [26]. In this tool, we include a FAST5 “signal” mode which generates interactive plots of raw pore signal intensity and corresponding base calls.

### 3.5 POD5

The recently released ONT POD5 format is a more efficient data format designed to replace FAST5 [27]. It differs from FAST5 in that it does not contain sequence information: The basecalled sequence for POD5 files is stored in a separate BAM file. LongReadSum can process the POD5 together with its corresponding basecalled BAM file (e.g. from ONT Dorado [17]) to generate the signal-to-base correspondence plots.

## 4 Results

### 4.1 Basic usage for QC summary

Here we show an example BAM file QC output for an ONT whole-genome sequencing (WGS) dataset of the HG002 human genome sequenced with the ONT PromethION R10.4.1 platform, base called with ONT Guppy v6.3.8 and aligned to the GRCh37 reference. The HTML report includes a table of basic statistics for mapped and unmapped sequencing reads and bases including the N50, a useful statistical measure for describing read length and quality, as well as GC content, a useful indicator of DNA thermostability (Fig. 1A). The report also includes interactive histograms for read lengths and base quality scores reported by the sequencing platforms (Fig. 1B, C). Read length histograms help identify issues with sample preparation or sequencing which may result in reads of unexpected lengths (Fig. 1B). Expected read length distributions vary from ∼8–50 kb as expected based on the sample preparation [28]. A histogram of base quality scores reported by the basecaller (Fig. 1C) as well as a histogram of read quality scores (Fig. 1D) can be used to identify biases in the sequencing run, such as whether the sequencing quality is generally consistent across reads, and any potential errors which may affect downstream analyses. For instance, in ONT sequencing, lower base quality scores reported by the basecaller can arise from the presence of modified bases that the basecaller may not have seen during the training. In basecalls for this sample generated by Guppy v6.3.8, most base quality Phred scores are > 20, indicating high confidence calls. In this sample, the median per-read GC content is 41 % (Figs. 1A, 1E), which is the expected value for HG002 [29]. Similar to FastQC, we add QC flags to the LongReadSum-generated HTML report, and a “warning” icon next to a specific QC section indicates a flag in the corresponding section.

### 4.2 Use cases for different data types

Here we demonstrate different data type use cases, including analysis of cDNA sequencing, direct mRNA sequencing, ONT reduced representation methylation sequencing (RRMS), ONT whole-genome sequencing with base modification detection, as well as for whole genome sequencing (WGS) in general.

**Fig. 2:**
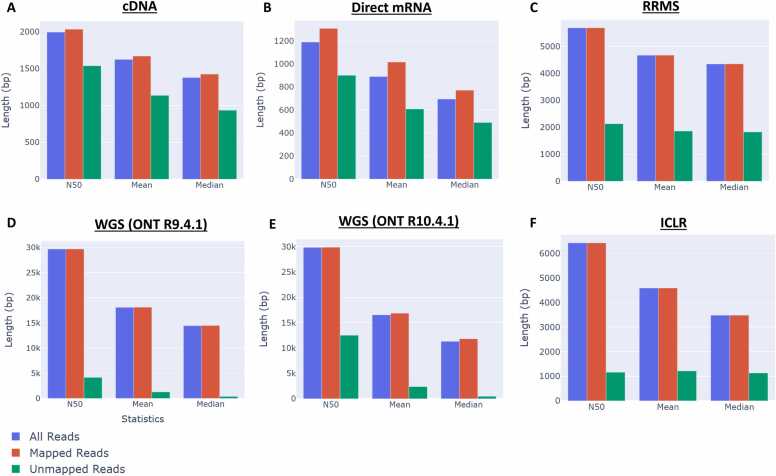
**Comparison of read lengths for different types of data.** (A) cDNA. (B) Direct RNA. (C) Reduced representation methylation sequencing (RRMS). QC for accepted reads passing RRMS filtering steps are shown. (D) Whole-genome sequencing (WGS) with ONT R9.4.1 MinION flowcells. (E) WGS with ONT R10.4.1 PromethION flowcells. (F) WGS with Illumina Complete Long Read (ICLR) sequencing.

Both cDNA and direct RNA sequencing on the ONT platform involve the sequencing of smaller molecules that fall within several kilobases. This can be seen by comparing read length distributions for the K562 cell line cDNA sequenced using MinION R9.4.1 flowcells (Fig. 2A) and the RNA sequenced using PromethION R9.4.1 flowcells (Fig. 2B). When using reduced representation methylation sequencing (RRMS) on a DNA sample, a read length N50 of ∼5.5 kb is shown (Fig. 2C). RRMS is a methylation detection approach that leverages adaptive sampling to enrich key genomic regions during sequencing. In a different DNA sample, ONT WGS library preparation resulted in a read length N50 of ∼30 kb across two flowcell types (Fig. 2D, E). The HG002 Illumina Complete Long Read (ICLR) sequencing data exhibits an expected N50 of ∼6.5 kb (Fig. 2F) [2].

**Fig. 3:**
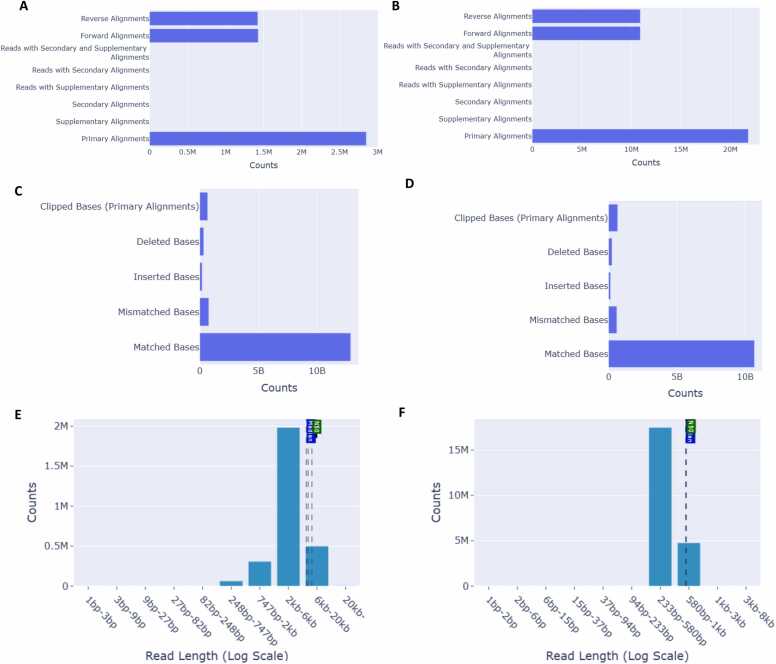
Comparison of accepted vs. rejected read statistics for RRMS data from the COLO829 cell line. RRMS read alignment types for accepted (A) vs. rejected (B) reads; Base alignment counts for accepted (C) vs. rejected (D) reads; RRMS read length histogram in log scale for accepted (E) vs. rejected (F) reads.

LongReadSum also uses the resulting CSV file from RRMS to produce a separate QC report for accepted reads that pass the RRMS filtering criteria, as well as for rejected reads. In Fig. 3, we show QC for accepted vs. rejected reads for RRMS data on the COLO829 cell line (Figs. 3A, 3B). The base alignment error rates for accepted vs. rejected reads are largely similar (Figs. 3C, 3D). Accepted RRMS fragment sizes are much larger than rejected reads, and this can be seen by comparing the two read length histograms (Figs. 3E, 3F).

**Fig. 4:**
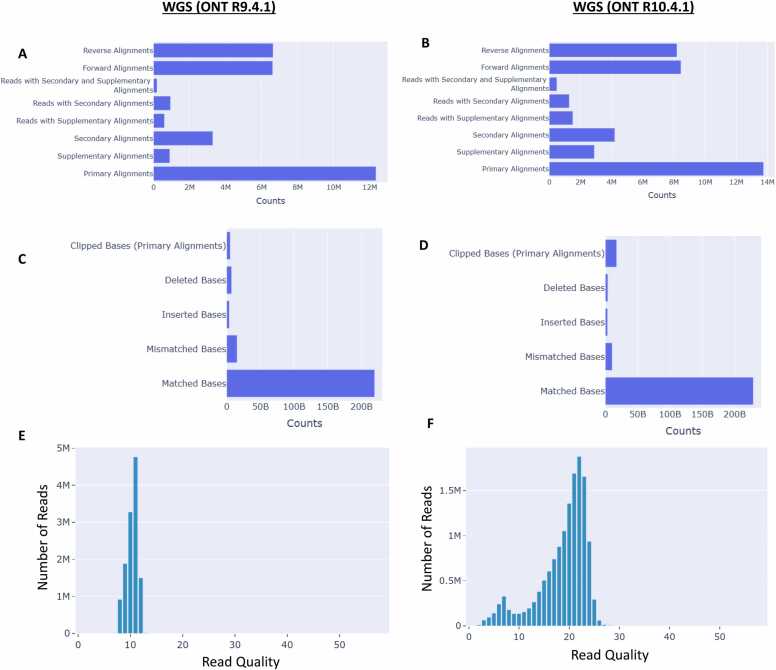
Comparison of alignment error rates and base quality scores in whole-genome sequencing for ONT R9.4.1 MinION vs. R10.4.1 PromethION flowcells on the HG002 sample. (A) ONT R9.4.1 read alignment counts. (B) R10.4.1 read alignment counts. (C) ONT R9.4.1 base alignment error counts. (D) R10.4.1 base alignment error counts. (E) ONT R9.4.1 read quality distributions. (F) R10.4.1 read quality distributions.

We use data from the HG002 sample to show that the ONT R10.4.1 flow cell improves upon read accuracy when compared to R9.4.1. Although the R10.4.1 data was sequenced using PromethION, which produces over ∼6 times more sequencing data per flow cell than the MinION with the R9.4.1 flowcell [30], we can visualize the increase in sequencing read accuracy with R10.4.1 by comparing read average base quality distributions as well as the percentage of total mapped bases with mismatches, insertions, and deletions (Fig. 4). LongReadSum provides metrics for each type of read and base alignment. In general, higher read and base alignment rates are indicative of high-quality sequencing data with lower error rates.

**Fig. 5:**
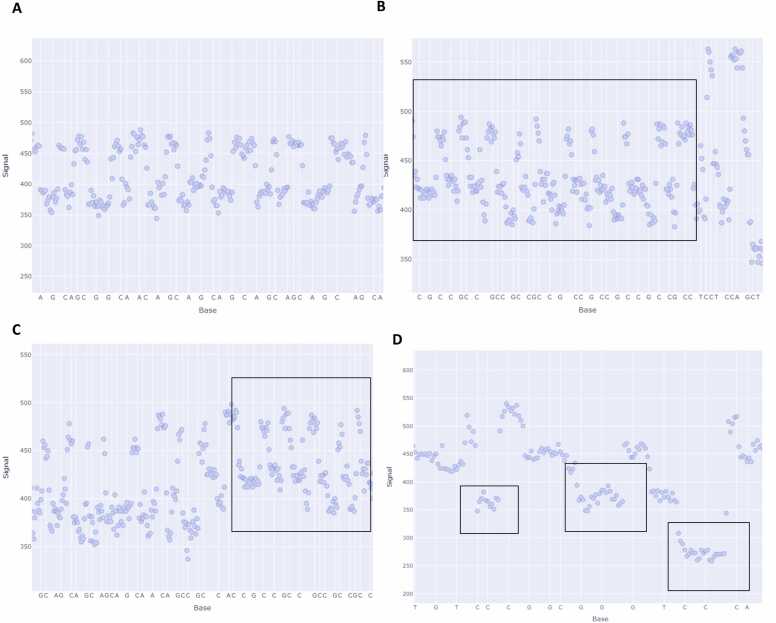
Repeat patterns in the FAST5 file ionic current signal data from ONT whole-genome sequencing are observable using LongReadSum. Specific repeat regions are indicated in the black box. (A) [CAG]n tandem repeats; (B,C) [GCC]n tandem repeats; (D) [C]3 and [G]3 repeats.

Next, we show how signal information from a FAST5 (or POD5) file can be used to validate the presence of regions of interest (Fig. 5). FAST5 and POD5 files contain raw signal information generated during sequencing. This information is used for base calling and can be stored for future analysis with improved base calling algorithms. Thus, the signal data is highly valuable and can also be used to identify specific patterns in regions of interest. For example, repeat motifs including [CAG]n (Fig. 5A), [GCC]n (Figs. 5B, 5C), as well as [C]n and [G]n (Fig. 5D) each exhibit recurring signal patterns which can serve as evidence for validating the presence of repeat regions. Using LongReadSum, it is possible to visually examine these read signal patterns to identify possible short tandem repeats.

In Supplementary Fig. 2, we show an example output for a POD5 file from HG002 sequenced with ONT R10.4.1, and its corresponding BAM file basecalled with Dorado [17]. While the POD5 file contains the raw signal data, the BAM file contains the sequence and basecalling information that allows us to produce the signal-to-sequence correspondence plots for each read.

To show the ability to summarize methylation calls, in Supplementary Table 1, we show an example LongReadSum base modification summary output for the HG002 sample sequenced with MinION R9.4.1, basecalled with Guppy version 5.0.1, aligned to the GRCh38 reference genome, and with 5-methylcytosine (5mC) detection. We compare our results with modkit version 0.3.1, an ONT tool that provides similar metrics, generating either a summary report of read-level statistics from a subsample of reads, or a more granular summary of per-site base modifications which requires a reference genome [11]. LongReadSum provides information including total number of predictions, total predictions exceeding the user-specified modification probability threshold, total aligning to CpG sites in the reference genome, as well as read length percentage vs. base modification probability for each type of modification. We compare results with modkit by running the following command, which yields CpG per-site modification values for a base modification probability threshold of 0.8:

*modkit pileup $input_bam $output_txt --ref $ref_genome --filter-threshold 0.8 –cpg*.

We then use a custom Python script to parse the modkit output file, which contains a list of unique reference positions with modifications, strand, and the number of reads supporting modification predictions (N_mod_). Our resulting counts for modified CpG sites in the forward and reverse strand are identical to the modkit results obtained by summing over all values of N_mod_ for each strand. Furthermore, we show an example of the LongReadSum summary of base modification probability distributions calculated from ONT whole-genome sequencing on the HG002 sample. The sample is sequenced with ONT R9.4.1 MinION with 5mC base modification detection using ONT Guppy and DeepMod2 (Supplementary Fig. 1).

For RNA-Seq BAM files, the user can specify a corresponding BED12 file with gene annotations, and LongReadSum will generate a TSV of TIN scores for each transcript. The mean, median, and standard deviation of TIN scores for the sample will be saved in a summary text file and is also included as a table in the HTML report. In Supplementary Table 2, we show a comparison of LongReadSum vs. RSeQC TIN score summaries for an RNA-Seq BAM file (9.92 gigabases, with an N50 of 1.1 kilobases) of the GTEX-14BMU lung sample from the GTEx V9 Long Read RNA-Seq Data release of ONT-sequenced human mRNA samples [31], using the GENCODE Release 46 basic gene annotations on the reference chromosomes (converted from GTF to BED12 format) [32]. Like RSeQC, LongReadSum includes a sample size parameter, which is the number of equal-spaced nucleotide positions picked from mRNA to improve computational performance, as well as the minimum coverage parameter, which represents the minimum number of reads mapped to a transcript.

LongReadSum uses the HTSLib C++ library [33] to extract all reads overlapping the transcript region, filtering out reads flagged as unmapped, secondary, duplicates, or reads with QC failure. The CIGAR strings for each resulting read are then parsed to count alignments at each nucleotide position (matches and mismatches), skipping overlapping sequences, null bases, and bases with a Phred score less than 13. While RSeQC implements the same approach, there is an occasional small discrepancy in the number of reads for each transcript that we believe is due to minor implementation differences between the two tools. For this sample, the median TIN is > 70, which generally indicates low RNA degradation [19].

### 4.3 Performance

We compared the performance of LongReadSum with other widely used QC tools with similar applications and summarized results in Supplementary Table 3. We evaluate performance for summarizing QC metrics for an 18 GB FASTQ file of passing reads from a single sequencing run of the ONT HG002 5mC dataset. For NanoPlot [7], NanoQC [7], and FASTQC [6], which provide similar information in HTML reports, LongReadSum utilizes half the CPU and wall clock time, while FASTQC and NanoQC utilize significantly more memory. NanoQ [9] exhibits the lowest overall CPU, wall clock time, and maximum memory usage. This is expected since NanoQ is a minimal QC tool that yields only a summary text report.

For evaluating BAM QC performance, we use the full 376 GB HG002 ONT R10.4.1 whole genome dataset. LongReadSum has a comparable wall clock time with NanoPlot but with half the CPU time and with less memory, indicating a more efficient use of computational resources. FASTQC BAM analysis completes in ∼4 hours of CPU and wall clock time with ∼56 GB maximum memory usage, significantly higher than LongReadSum and NanoPlot, which both also provide more comprehensive long-read metrics than FASTQC.

For evaluating performance on base modification analysis, we use an 11 GB sequencing run from the ONT R9.4.1 HG002 5mC dataset. We compare performance with ONT Modkit [34], a widely used tool for obtaining comprehensive summaries of modification statistics. LongReadSum uses significantly less maximum memory (<1 GB for LongReadSum vs. ∼43 GB for Modkit) and less CPU time, but with increased wall clock time (2 hours 40 minutes for LongReadSum, and 54 minutes for Modkit). While LongReadSum and Modkit both provide a summary of modification counts, they also generate distinct metrics which can explain these discrepancies. For example, Modkit also generates per-site modification information, while LongReadSum also generates a full summary of the BAM sequence and alignment statistics.

For RNA-Seq TIN analysis, we use the 16 GB RNA-Seq BAM file of the GTEX-14BMU lung sample and compare LongReadSum performance with RSeQC [20]. LongReadSum utilizes significantly more CPU, wall clock time, and memory. We are currently working on improving the efficiency of our own implementation to achieve comparable performance with RSeQC.

## 5 Discussion

LongReadSum is a fast and versatile tool providing a comprehensive overview of long read sequencing and alignment information, enabling the identification of significant errors that may affect downstream analyses. It supports all major sequencing data formats, such as FASTA, FASTQ, and BAM, ONT FAST5, ONT POD5, ONT basecall summary files, and PacBio unaligned BAM files and Illumina Complete Long Read (ICLR) FASTQ file formats. It allows for quick identification of quality issues and biases in read and base quality distributions, alignments, base modifications, and RNA-Seq data prior to downstream analyses. It also offers the ability to analyze Oxford Nanopore ionic current signal data and base calls in specific genomic regions.

In the future we aim to continue developing LongReadSum to support evolving standards and file formats, as well as to provide support for emerging technologies often used in conjunction with long read sequencing, such as optical mapping. BioNano optical maps have important applications in genomic analysis pipelines, including for *de novo* assemblies and structural variant calling. Thus, we plan to add QC support for optical map file formats including BNX and CMAP.

Finally, standards for sequencing file formats are continuously evolving with improvements in size, accuracy, and read/write speed to streamline downstream analyses. Although keeping up with evolving standards is a challenging task, we welcome these opportunities to expand our QC and summarization tool for the benefit of future users by incorporating support for the latest high-performance file formats. In the future we also plan to include more in-depth mapping and base quality QC statistics, and support for summarizing multiple sequencing runs on the same sample or multiplexed samples in the same run. We hope that this tool is a useful addition to the community, in particular for researchers analyzing large sequencing datasets across multiple platforms. LongReadSum is released under an MIT license and is available at https://github.com/WGLab/LongReadSum.

## CRediT authorship contribution statement

**Perdomo Jonathan Elliot:** Writing – review & editing, Writing – original draft, Software, Funding acquisition. **Fang Li:** Software. **Wang Kai:** Writing – review & editing, Supervision, Funding acquisition, Conceptualization. **Ahsan Mian Umair:** Writing – review & editing, Software. **Liu Qian:** Software.

## Declaration of Competing Interest

The authors declare that they have no known competing financial interests or personal relationships that could have appeared to influence the work reported in this paper.
