# Supplementary material for: LongReadSum: A fast and flexible quality control and signal summarization tool for long-read sequencing data
Source: Comput Struct Biotechnol J. 2025 Jan 24;27:556–63. doi: 10.1016/j.csbj.2025.01.019 (PMC11840941; doi:10.1016/j.csbj.2025.01.019)
Supplement: Supplementary file 1 — Supplementary material [file mmc1.docx]

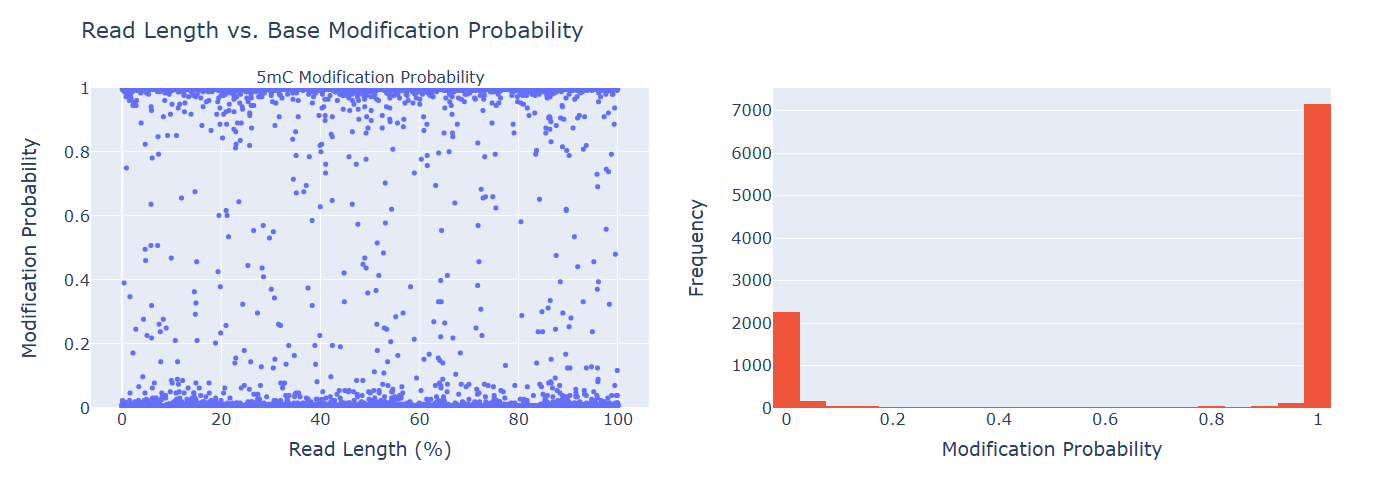


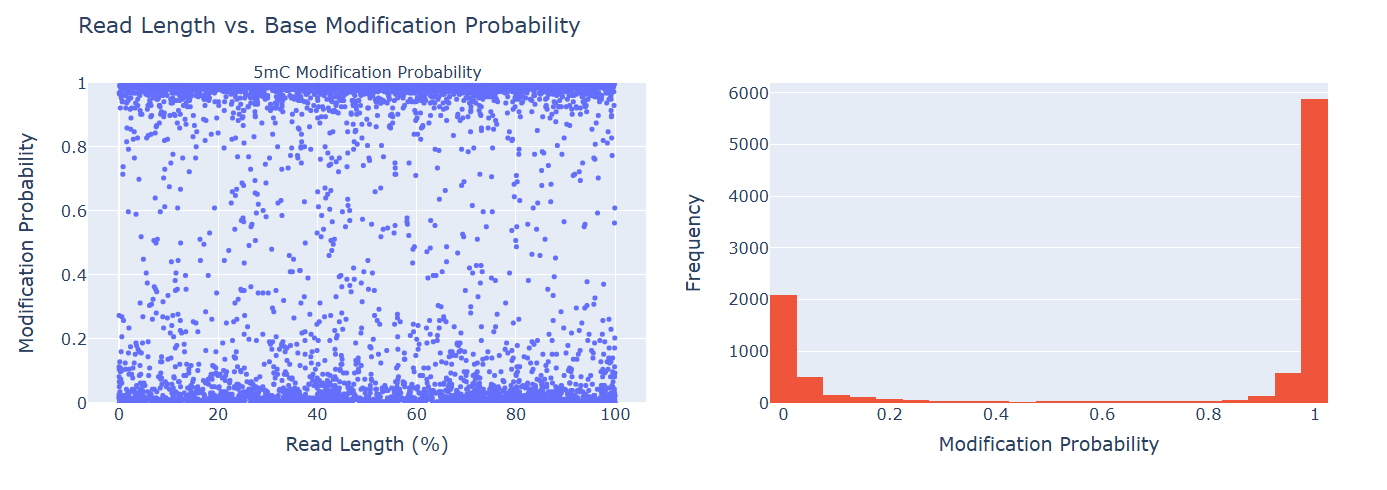


Supplementary Figure 1. LongReadSum summary of base modification probability distributions calculated from ONT whole-genome sequencing on the HG002 sample. The sample is sequenced with ONT R9.4.1 MinION with 5mC base modification detection using ONT Guppy (upper panel) and DeepMod2 (lower panel).


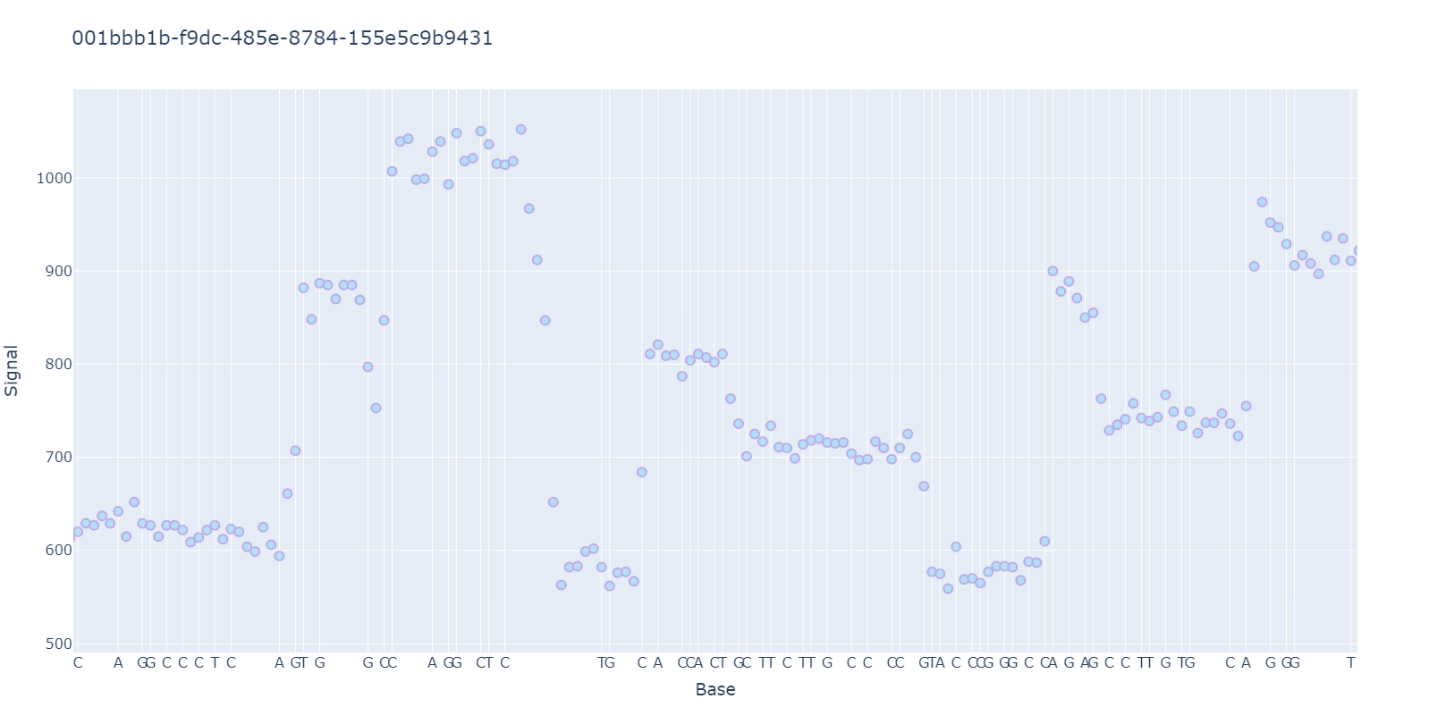


Supplementary Figure 2. POD5 + basecalled BAM file ionic current signal data for a single read from ONT whole-genome sequencing are observable using LongReadSum. Data is HG002 sequenced with ONT R10.4.1.

*
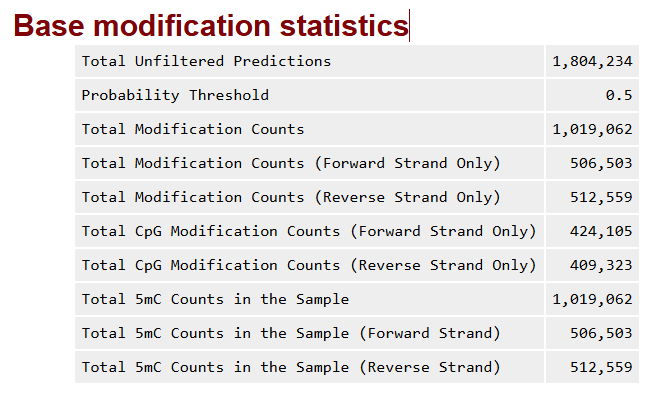
*

*Supplementary Table 1. LongReadSum summary base modification and CpG site statistics from ONT whole-genome sequencing. Data is HG002 sequenced with ONT R9.4.1 MinION and 5mC base modification detection.*

| TIN Summary | LongReadSum | RSeQC |
| --- | --- | --- |
| **Number of transcripts** | 67,069 | 67,069 |
| **Median TIN** | 74.250 | 74.256 |

Supplementary Table 2. Comparison of LongReadSum vs. RSeQC TIN score summary results for a single ONT long-read GTEx RNA-Seq BAM file (9.92 gigabases, with an N50 of 1.1 kilobases) using a TIN sample size of 100, and a minimum coverage of 2.

| FASTQ QC Performance | LongReadSum | NanoPlot | NanoQ | NanoQC | FASTQC |
| --- | --- | --- | --- | --- | --- |
| **Wall clock time (H:M:S)** | 0:03:16 | 0:09:37 | 0:02:31 | 0:08:55 | 0:06:41 |
| **CPU time (H:M:S)** | 0:03:11 | 0:09:30 | 0:01:45 | 0:08:53 | 0:06:41 |
| **Max. memory usage (GB)** | 0.33 GB | 0.23 GB | 0.02 GB | 2.03 GB | 2.97 GB |

| BAM QC Performance | LongReadSum | NanoPlot | FASTQC |
| --- | --- | --- | --- |
| **Wall clock time (H:M:S)** | 0:37:42 | 0:43:53 | 4:07:49 |
| **CPU time (H:M:S)** | 1:28:01 | 4:53:17 | 4:06:11 |
| **Max. memory usage (GB)** | 8.98 GB | 9.80 GB | 55.76 GB |

| Base modification QC Performance | LongReadSum | Modkit |
| --- | --- | --- |
| **Wall clock time (H:M:S)** | 2:40:30 | 0:54:51 |
| **CPU time (H:M:S)** | 2:39:43 | 3:25:15 |
| **Max. memory usage (GB)** | 0.96 GB | 43.31 GB |

| RNA-Seq TIN  QC Performance | LongReadSum | RSeQC |
| --- | --- | --- |
| **Wall clock time (H:M:S)** | 31:30:09 | 7:18:51 |
| **CPU time (H:M:S)** | 31:31:00 | 7:17:13 |
| **Max. memory usage (GB)** | 8.82 GB | 0.59 GB |

Supplementary Table 3. Comparison of LongReadSum CPU, wall clock time, and memory efficiency versus other tools for different applications (FASTQ QC, mapped bam QC, base modification QC, and RNA-Seq TIN scores). All results were generated using a 12-core compute node with 100GB memory. The following is a list of the software versions used with default parameters: NanoPlot v1.43.0, NanoQ v0.10.0, NanoQC v0.10.0, FASTQC v0.12.1 (non-interactive mode), Modkit v0.3.1 (pileup mode), RSeQC v5.0.2 (tin.py). For FASTQ analysis, we use a combined 18GB FASTQ of passing reads from a single sequencing run of the ONT HG002 5mC dataset. For BAM analysis, we use the full 376GB HG002 ONT R10.4.1 whole genome dataset. For base modification analysis, we use an 11GB sequencing run from the ONT R9.4.1 HG002 5mC dataset. For RNA-Seq TIN analysis, we use a 16GB GTEx v9 sample sequenced with ONT RNA-seq.
